# The serum soluble ASGR1 concentration is elevated in patients with coronary artery disease and is associated with inflammatory markers

**DOI:** 10.1186/s12944-024-02054-8

**Published:** 2024-03-27

**Authors:** Qin Luo, Jingfei Chen, Yanfeng Yi, Panyun Wu, Yingjie Su, Zhangling Chen, Hacı Ahmet Aydemir, Jianjun Tang, Zhenfei Fang, Fei Luo

**Affiliations:** 1 Department of Cardiovascular Medicine, The Second Xiangya Hospital, Central South University, Changsha, 410011 Hunan China; 2 Research Institute of Blood Lipids and Atherosclerosis, the Second Xiangya Hospital, Central South University, Changsha, China; 3 Reproductive Medicine Center, Department of Obstetrics and Gynecology, The Second Xiangya Hospital, Central South University, Changsha, Hunan China; 4 Department of Emergency Medicine, Hengyang Medical School, The Affiliated Changsha Central Hospital, University of South China, Changsha, China; 5 Kayakyolu Family Health Center, 33 Palandöken, Erzurum, Turkey

**Keywords:** Soluble asialoglycoprotein receptor 1, Lipid metabolism, Coronary artery disease, High-sensitivity C-reactive protein, White blood cell count, Inflammation

## Abstract

**Background and aims:**

Current research has suggested that asialoglycoprotein receptor 1 (ASGR1) is involved in cholesterol metabolism and is also related to systemic inflammation. This study aimed to assess the correlation between the serum soluble ASGR1 (sASGR1) concentration and inflammatory marker levels. Moreover, the second objective of the study was to assess the association between sASGR1 levels and the presence of coronary artery disease (CAD).

**Methods:**

The study subjects included 160 patients who underwent coronary angiography. Ninety patients were diagnosed with CAD, while seventy age- and sex-matched non-CAD patients served as controls. We measured the serum sASGR1 levels using an ELISA kit after collecting clinical baseline characteristics.

**Results:**

Patients with CAD had higher serum sASGR1 levels than non-CAD patients did (*P* < 0.0001). sASGR1 was independently correlated with the risk of CAD after adjusting for confounding variables (OR = 1.522, *P* = 0.012). The receiver operating characteristic (ROC) curve showed that sASGR1 had a larger area under the curve (AUC) than did the conventional biomarkers apolipoprotein B (APO-B) and low-density lipoprotein cholesterol (LDL-C). In addition, multivariate linear regression models revealed that sASGR1 is independently and positively correlated with high-sensitivity C-reactive protein (CRP) (*β* = 0.86, *P* < 0.001) and WBC (*β* = 0.13, *P* = 0.004) counts even after adjusting for lipid parameters. According to our subgroup analysis, this relationship existed only for CAD patients.

**Conclusion:**

Our research demonstrated the link between CAD and sASGR1 levels, suggesting that sASGR1 may be an independent risk factor for CAD. In addition, this study provides a reference for revealing the potential role of sASGR1 in the inflammation of atherosclerosis.

## Introduction

Atherosclerotic cardiovascular disease (ASCVD) is a fatal disease with a complex etiology worldwide. Over the last few decades, there has been constant updating and even subversion of the understanding of atherosclerosis [1]. In parallel, an increasing number of atherosclerotic markers have been discovered [2, 3]. Disorders of lipid metabolism, especially hypercholesterolemia, are well-known pathogenic risk factors for ASCVD [4]. Lipoproteins are the initial points of interest. Oxidized low-density lipoprotein (ox-LDL) or small dense LDL enters the artery wall, which constitutes the classic early stage of atherosclerosis [2, 5]. Of course, circulating adhesion molecules such as vascular cell adhesion molecule-1 (VCAM), intercellular adhesion molecule-1 (ICAM), and monocyte chemotactic protein-1 (MCP-1) play crucial roles in this stage [5, 6]. High triglyceride (TG) and residual cholesterol levels are important pathogenic factors of ASCVD and were recognized when residual cardiovascular risk associated with statins was discovered [7, 8]. Other lipoproteins, such as lipoprotein(a) [Lp(a)] and apolipoprotein B (APO-B), have also been found to be associated with the risk of atherosclerosis [5]. Evaluating the characteristics of coronary artery plaques using the latest invasive or noninvasive imaging methods can help predict the risk of cardiac events and guide personalized treatment strategies [9–11]. A higher plaque burden and high risk plaques are independent risk factors for myocardial ischemia [11–13]. In addition, recent studies have established that inflammatory markers participate in all stages of atherosclerosis, making them one of the most promising therapeutic targets for treating this disease [14, 15].

Asialoglycoprotein receptor (ASGR) is a hepatic C-type lectin that is expressed mainly on the sinusoidal surface of hepatocytes [16, 17]. The primary function of ASGR is to bind glycoproteins containing terminal galactose or GlcNAc residues (such as asialoglycoproteins) in circulation [18, 19]. The complex is subsequently internalized under the coat of clathrin and transported to the lysosome for degradation [20]. As a hepatocyte membrane receptor, asialoglycoprotein receptor 1 (ASGR1) regulates hepatic cholesterol homeostasis by interacting with circulating asialoglycoproteins [21]. In animal studies, inhibiting hepatic ASGR1 or its binding to circulating asialoglycoproteins reduces serum cholesterol levels by promoting cholesterol efflux into the bile [21]. *ASGR1* loss-of-function mutations are linked to a 0.4 mmol/l decrease in circulating non-high-density lipoprotein cholesterol (non-HDL-C) and a 34% reduction in the incidence of coronary artery disease (CAD) [22]. Notably, the effect of the *ASGR1* mutation on the risk of CAD is significantly greater than that on non-HDL-C levels, suggesting that the significant reduction in the risk of CAD is not entirely explained by the effect of ASGR1 mutations on non-HDL-C levels [22, 23]. Therefore, *ASGR1* mutations may be involved in other protective mechanisms than the regulation of cholesterol homeostasis, such as inflammation. Indeed, some direct or indirect evidence has shown that ASGR1 is involved in biological processes related to systemic inflammation and vascular inflammation [24–27]. For example, proinflammatory cytokines upregulate the expression of ASGR1 [24]. The interaction of ASGR1 with epidermal growth factor receptor (EGFR) activates the extracellular signal-regulated kinase (ERK) pathway [25], which has been linked to inflammatory regulation and atherosclerosis [28, 29]. However, the relationship between ASGR1 and inflammation in patients with CAD is unclear.

In contrast to ASGR1, which is located on the liver surface mentioned above, serum soluble ASGR1 (sASGR1), which is secreted by the liver, is another splicing variant of liver ASGR1 and is located in the circulation due to the absence of a transmembrane domain [30]. However, current research on sASGR1 is very limited. Although our previous study showed that sASGR1 is associated with LDL-C levels [31], the relationship between sASGR1 levels and the risk of CAD is still unclear.

C-reactive protein (CRP) levels and white blood cell (WBC) counts are the most basic and widely used indicators of systemic inflammation. In addition, CRP levels are positively correlated with the degree of coronary artery stenosis in patients with CAD [32]. Two recent studies have established that high-sensitivity C-reactive protein (hs-CRP) levels exhibit superior predictive power for future cardiovascular events and mortality risk when compared to circulating cholesterol levels in patients receiving statin therapy [33, 34]. Similarly, WBC counts have been widely confirmed to be closely related to atherosclerosis and CAD [35–37]. Currently, there are no simple or effective methods for determining the level of ASGR1 expression on the hepatocyte membrane in clinical practice. Therefore, the purpose of this study was to assess the correlation between sASGR1 levels and inflammatory marker levels, including hs-CRP levels, WBC counts, and WBC subsets. Moreover, the second objective of the study was to assess the association between sASGR1 levels and the presence of CAD.

## Materials and methods

### Study population

We consecutively included 160 patients admitted to the Second Xiangya Hospital of Central South University between September 2022 and September 2023 in this study. All patients underwent coronary angiography. Patients were categorized into two groups based on the results of coronary angiography: 90 patients with CAD and 70 patients without CAD. We matched the groups for age and sex. Four experienced angiographers performed coronary angiography, two of whom evaluated the vessels. To determine the severity of CAD, CAD patients were further subdivided into acute myocardial infarction (AMI) (*n* = 26) and CAD without AMI (*n* = 64) groups. The severity of coronary lesions was determined by the number of major coronary artery stenoses, which were classified as vessels ≤ 2 (*n* = 51) or vessels > 2 (*n* = 39). Furthermore, the Gensini score was also calculated to assess the severity of CAD. We excluded patients with infections, autoimmune diseases, liver diseases, renal failure, malignancies, or other serious diseases.

The diagnosis of CAD was based on angina pectoris manifestations, electrocardiogram changes, and coronary angiography, which indicated that the major vessel had a degree of stenosis greater than or equal to 50%. Elevated plasma troponin levels, together with evidence of acute myocardial ischemia, are diagnostic criteria for AMI [38]. Fasting blood glucose levels ≥ 126 mg/dl (7.0 mmol/L) or 2-hour postprandial blood glucose levels ≥ 200 mg/dL (11.1 mmol/L) were used to diagnose type 2 diabetes mellitus (T2DM). Repeated measures of blood pressure greater than or equal to 140/90 mmHg were used to diagnose hypertension.

The study was authorized by the ethics committee of Second Xiangya Hospital, which also found that it adhered to the ethical principles of the Declaration of Helsinki. Informed permission was obtained from all patients.

### Sample size calculation

In the study assessing the association between sASGR1 levels and the presence of coronary artery disease (CAD), we used Power Analysis and Sample Size (PASS) software for sample size calculations. We set bilateral α = 0.05 and power = 0.9. The ratio of the sample size between the CAD group and the control group was 1.28:1. We set the mean and standard deviation of the sASGR1 levels for the two groups based on our preliminary results. The sample size calculation revealed that a sample of 55 CAD patients and 43 non-CAD patients achieved 90.31% power to reject the null hypothesis of equal means when the population mean difference was µ1- µ2 = 4–2.1 = 1.9 with standard deviations of 4 for the CAD group and 1.3 for the non-CAD group. Ultimately, 90 CAD patients and 70 non-CAD patients composed the sample.

### Clinical characteristics and laboratory measurements

Basic information, including age, sex, body mass index (BMI), smoking status, and statin use, was collected and recorded. Peripheral blood samples were collected from patients through the elbow vein after they had fasted overnight. The serum was collected after centrifuging the blood sample for 10 min at 3,000 rpm and subsequently stored at -80 °C. Serum lipid parameters and hs-CRP levels were measured using a fully automated biochemical analyzer. WBCs, neutrophils, lymphocytes, and monocytes were counted using an automatic blood cell counter.

### ELISA for determining the sASGR1 concentration

The serum concentration of sASGR1 was determined using sandwich enzyme-linked immunosorbent assay (ELISA) kits (JL41965; Jianglai Biology, Shanghai). Two measurements were repeated per sample to reduce random variations. Both the intraplate and interplate coefficients of variation (CVs) were less than 10%, suggesting that the assay has good repeatability. The recovery rate and linearity of this reagent kit were 95% and 91%, respectively (with a dilution ratio of 1:2). The minimum detectable concentration of serum sASGR1 was 0.156 ng/mL.

### Statistical analyses

For the data analysis, we employed SPSS 25.0 statistical software. In addition, EmpowerStats statistical software (version 4.1) and R language (version 4.2.0) were used for subgroup analysis and interaction testing. *P* = 0.05 was used as the statistical criterion.

The continuous variables are displayed as the mean ± standard deviation or as medians and quartiles (Q1-Q3), and group comparisons were conducted using the independent-samples t test or Mann‒Whitney U test according to the type of data distribution. Chi-square tests were used to compare differences among categorical variables, which are presented as frequencies or percentages. The data were analyzed for a normal distribution by the D’Agostino–Pearson omnibus normality test. The Kruskal‒Wallis test was used to assess the association between sASGR1 levels and the severity of CAD. A multivariate logistic regression model was used to identify the factors that influence the presence of CAD. The diagnostic value of sASGR1 and traditional biomarkers in patients with CAD was assessed using receiver operating characteristic (ROC) curves. To further analyze the relationship between sASGR1 and inflammatory markers, we employed a stepwise multivariate regression model in which inflammatory markers were used as the dependent variables. For subgroup analysis, stratified linear regression models were employed, and likelihood ratio tests were applied to find any variations or interactions.

## Results

### Baseline characteristics

The basic details and biochemical characteristics of the study subjects are listed in Table 1. BMI, smoking status, statin use, history of hypertension, and T2DM status were greater in the CAD group than in the non-CAD group (*P* < 0.05). The CAD group had higher serum TG, LDL-C, Lp(a), APO-B, and hs-CRP levels; WBC and neutrophil counts; and monocyte counts than did the non-CAD group (*P* < 0.05). HDL-C levels were greater in non-CAD patients (*P* < 0.05). There were no significant differences in age, sex, lymphocyte count, or other blood lipid parameters between the two patient groups.

**Table 1:** Baseline characteristics of the study participants

Variable	CAD (*n* = 90)	Non-CAD (*n* = 70)	*P* value
Age (year)	56.33 ± 8.70	55.79 ± 9.46	0.686
Sex (male, %)	52 (57.8%)	32 (45.7%)	0.152
BMI (kg/m^2^)	24.68 ± 3.61	23.05 ± 3.15	0.003
Smoking, n (%)	38 (42.2%)	13 (18.6%)	0.002
Statin use, n (%)	56 (62.2%)	7 (10.0%)	< 0.001
Hypertension, n (%)	53 (58.9%)	17 (24.3%)	< 0.001
T2DM, n (%)	26 (28.9%)	9 (12.9%)	0.02
TG (mmol/L)	1.86 (1.3, 2.7)	1.00 (0.8, 1.5)	< 0.001
TC (mmol/L)	4.42 (3.9, 5.4)	4.31 (3.7, 4.9)	0.104
LDL-C (mmol/L)	3.07 ± 1.05	2.69 ± 0.67	0.010
HDL-C (mmol/L)	1.07 ± 0.28	1.22 ± 0.26	< 0.001
Lp(a) (mg/L)	189.70 (100.5, 482.5)	76.60 (45.5, 164.4)	< 0.001
APO-A1 (g/L)	1.02 ± 0.19	1.08 ± 0.16	0.052
APO-B (g/L)	0.84 (0.7, 1.1)	0.77 (0.6, 0.9)	0.022
Hs-CRP (mg/L)	2.96 (1.2, 10.3)	1.04 (0.5, 2.1)	< 0.001
WBC count (×10^9^/L)	7.09 (5.7, 8.6)	5.85 (5.0, 6.6)	< 0.001
Neutrophil count (×10^9^/L)	4.46 (3.5, 5.8)	3.46 (2.9, 4.0)	< 0.001
Monocyte count (×10^9^/L)	0.43 (0.3, 0.5)	0.33 (0.3, 0.4)	< 0.001
Lymphocyte count (×10^9^/L)	1.77 ± 0.64	1.80 ± 0.52	0.704

Continuous variables are expressed as the mean ± standard deviation or median (interquartile range); categorical variables are presented as numbers (percentages). Abbreviations: BMI, body mass index; TC, total cholesterol; TG, triglyceride; LDL-C, low-density lipoprotein cholesterol; HDL-C, high-density lipoprotein cholesterol; Lp(a), lipoprotein (a); APO-A1, apolipoprotein A1; APO-B, apolipoprotein B; Hs-CRP, high-sensitivity C-reactive protein; WBC, white blood cell; CAD, coronary artery disease; T2DM, type 2 diabetes mellitus

### Serum sASGR1 levels in the CAD and non-CAD groups

**Fig. 1:**
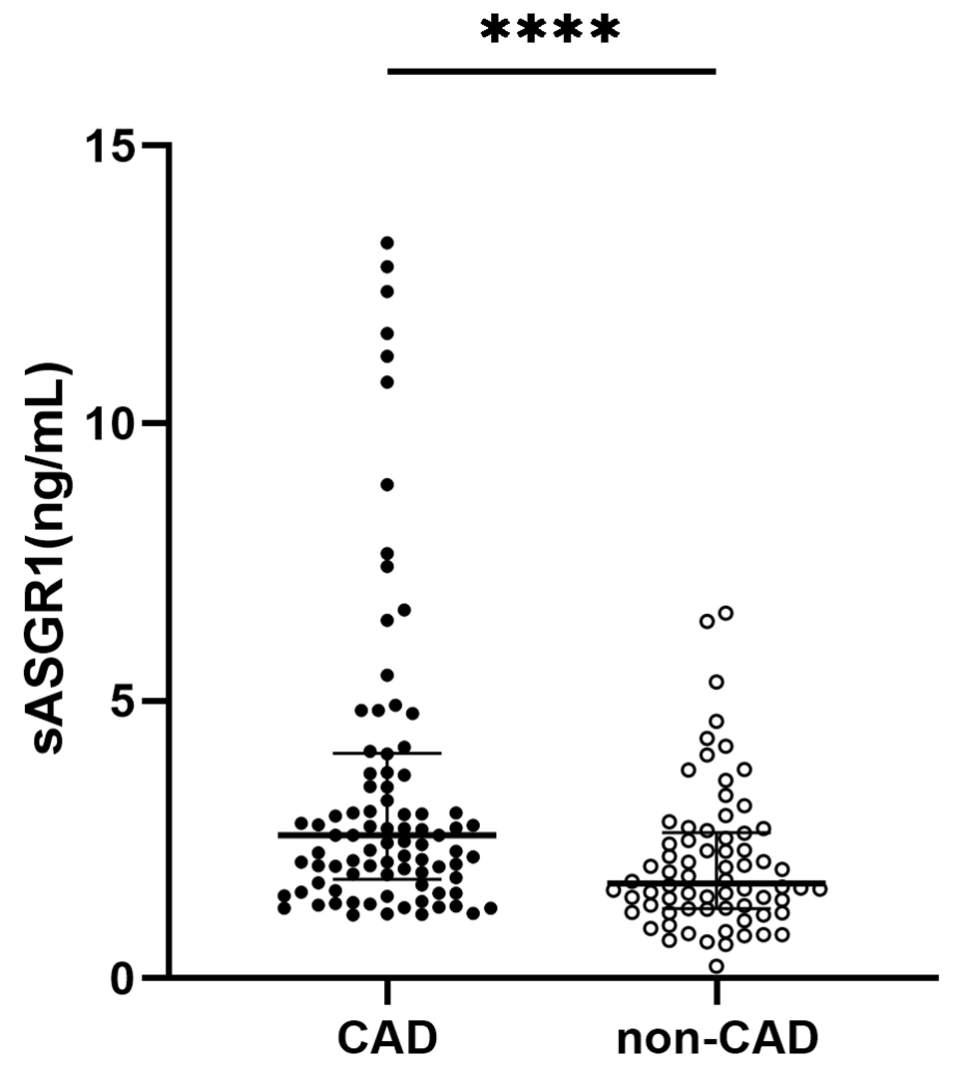
Comparison of serum sASGR1 levels between the CAD and non-CAD groups. The serum sASGR1 concentration in CAD patients (*n* = 90) was significantly greater than that in non-CAD patients (*n* = 70). [Mann‒Whitney U test, 2.58 (1.8, 4.1) vs. 1.71 (1.3, 2.6) ng/ml, *P* < 0.0001]. ****P < 0.0001

**Fig. 2:**
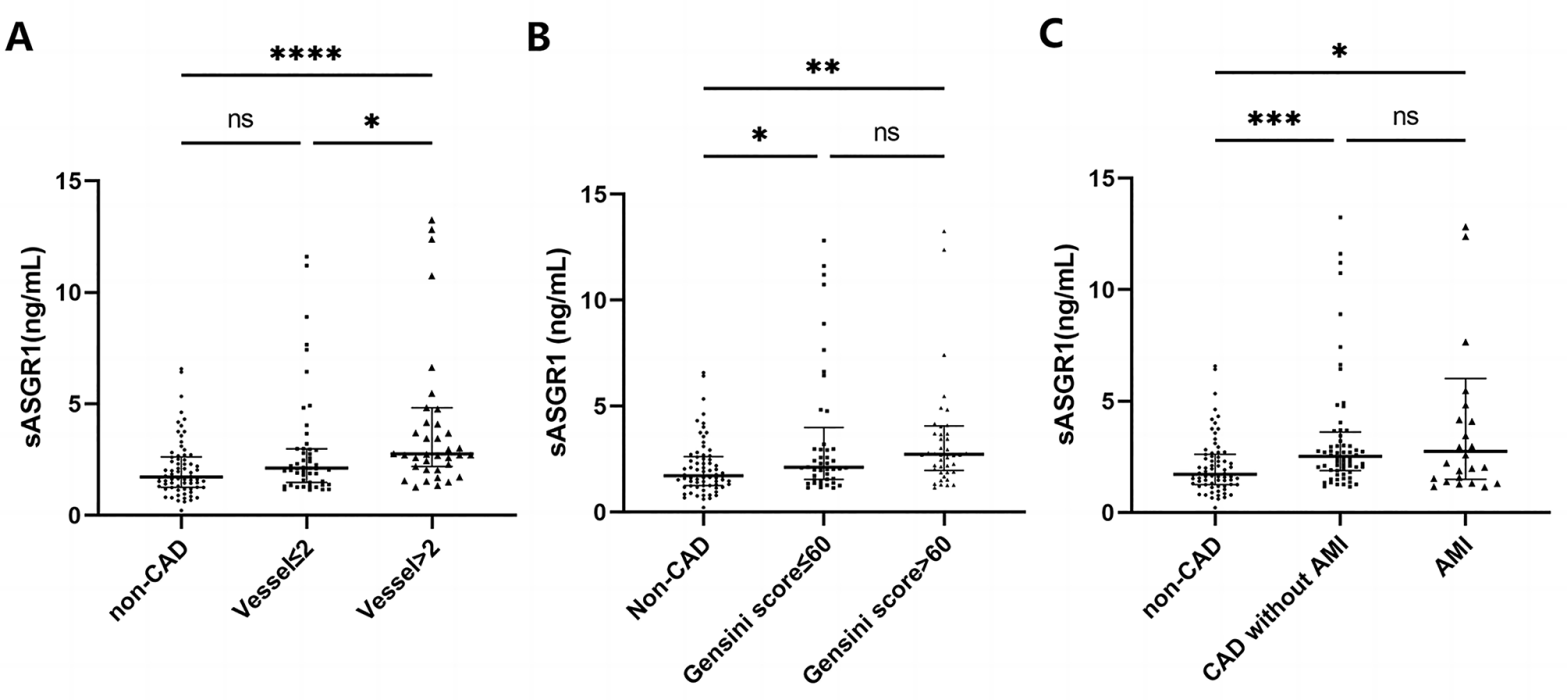
The relationship between sASGR1 levels and the severity of CAD. (**A**) sASGR1 levels in patients with different CAD statuses and numbers of involved vessels. Comparisons were evaluated by the Kruskal‒Wallis test. ns, not statistically significant. *P < 0.05. ****P < 0.0001. For vessels ≤ 2, the number of involved vessels was < 2; for vessels > 2, the number of involved vessels was more than 2. (**B**) Comparison of sASGR1 levels in patients with different Gensini scores. Comparisons were evaluated by the Kruskal‒Wallis test. ns, not statistically significant. *P < 0.05. **P < 0.01. (**C**) sASGR1 levels in patients with different severities of CAD. Comparisons were evaluated by the Kruskal‒Wallis test. ns, not statistically significant. *P < 0.05. ***P < 0.001

As shown in Fig. 1, the serum level of sASGR1 in CAD patients was significantly greater than that in non-CAD patients [2.58 (1.8, 4.1) vs. 1.71 (1.3, 2.6) ng/ml, *P* < 0.0001]. Subsequently, we investigated the relationship between sASGR1 levels and the severity of CAD. As shown in Fig. 2A, a lack of significant difference was observed between CAD patients with ≤ 2 diseased vessels and non-CAD patients [2.12 (1.5, 3.0) vs. 1.71 (1.3, 2.6) ng/ml, *P* = 0.078]. However, the level of sASGR1 in CAD patients with > 2 diseased vessels was significantly greater than that in non-CAD patients [2.76 (2.2, 4.8) vs. 1.71 (1.3, 2.6) ng/ml, *P* < 0.0001] or in CAD patients with ≤ 2 diseased vessels [2.76 (2.2, 4.8) vs. 2.12 (1.5, 3.0) ng/ml, *P* = 0.033]. Additionally, we separated patients into two groups according to the median (60) Gensini score to assess the association between sASGR1 level and CAD severity more precisely: Gensini score ≤ 60 (*n* = 45) and Gensini score > 60 (*n* = 45). However, there was no significant increase in the level of sASGR1 as the Gensini score increased (Fig. 2B). In addition, we were unable to identify significant differences in the severity of CAD between individuals with and without AMI (Fig. 2C).

### Diagnostic value of sASGR1 and traditional biomarkers for CAD patients

**Fig. 3:**
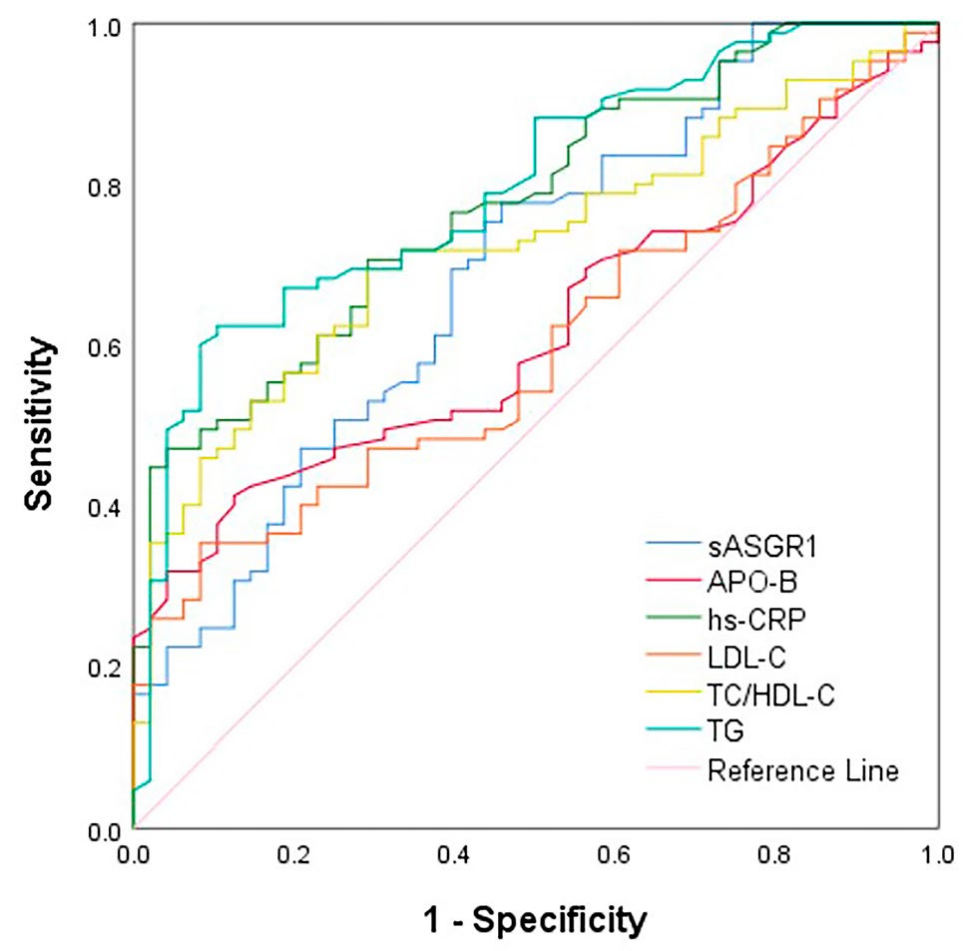
ROC curve analysis for the predictive value of serum sASGR1 and traditional biomarkers in the presence of CAD

We used receiver operating characteristic (ROC) curves to evaluate the diagnostic ability of sASGR1 levels for CAD (Fig. 3). The area under the curve (AUC) of sASGR1 was 0.691 (95% CI = 0.60–0.78, *P* < 0.001). Its diagnostic utility was better than that of the traditional biomarkers APO-B (AUC: 0.619, 95% CI: 0.53–0.71, *P* = 0.023) and LDL-C (AUC: 0.599, 95% CI: 0.50–0.69, *P* = 0.059), despite not being comparable to that of hs-CRP (AUC: 0.775, 95% CI: 0.70–0.85, *P* < 0.001), TG (AUC: 0.796, 95% CI: 0.72–0.87, *P* < 0.001), or total cholesterol (TC)/high-density lipoprotein cholesterol (HDL-C) (AUC: 0.723, 95% CI: 0.64–0.81, *P* < 0.001). sASGR1 has a cutoff value of 1.682 ng/ml for predicting the occurrence of CAD, with a sensitivity of 77.6% and a specificity of 54.2%. The sensitivity and specificity of the other biomarkers for predicting the presence of CAD were as follows: hs-CRP (sensitivity: 47.1%, specificity: 95.8%), APO-B (sensitivity: 41.2%, specificity: 87.5%), LDL-C (sensitivity: 35.3%, specificity: 91.7%), TC/HDL-C (sensitivity: 69.4%, specificity: 70.8%), and TG (sensitivity: 62.4%, specificity: 89.6%).

### sASGR1 is an independent influencing factor for the occurrence of CAD

A multivariate logistic regression model was used to identify the factors that influence the presence of CAD. We included independent variables based on the results of univariate analysis, clinical significance, sample size, and multicollinearity. The results showed that sASGR1, smoking, TG, hypertension, and T2DM were associated with an increased risk of CAD after controlling for BMI, smoking status, TG, LDL-C, hs-CRP, hypertension, and T2DM (Table 2). For every unit increase in the sASGR1 level, the risk of CAD increased by 0.52 times (OR = 1.522, 95% CI = 1.095, 2.115; *P* = 0.012).

**Table 2:** Multivariate logistic regression analysis of the influencing factors of CAD incidence

	β	OR (95% CI)	*P* value
sASGR1	0.420	1.522 (1.095, 2.115)	0.012
BMI	0.104	1.109 (0.944, 1.304)	0.209
Smoking	1.135	3.110 (1.066, 9.074)	0.038
TG	1.102	3.010 (1.535, 5.900)	0.001
LDL-C	0.012	1.012 (0.534, 1.917)	0.971
Hs-CRP	0.061	1.063 (0.932, 1.213)	0.362
Hypertension	1.760	5.811 (2.106, 16.036)	0.001
T2DM	1.574	4.825 (1.313, 17.738)	0.018

Abbreviations: CI, confidence interval. Other abbreviations are as shown in Table 1

### Relationships between the serum sASGR1 concentration and inflammatory marker level

By setting hs-CRP levels, WBC counts, and WBC subsets as dependent variables, we used stepwise multivariate regression models to determine the correlation between sASGR1 levels and inflammatory marker levels (Table 3). After we adjusted for sex, age, BMI, smoking status, statin use, hypertension status, T2DM status, and CAD status (Model II), sASGR1 was positively associated with the levels of inflammatory markers [hs-CRP (*β* = 1.00, *p* < 0.001), WBC count (*β* = 0.12, *P* = 0.004), and neutrophil count (*β* = 0.09, *P* = 0.011)]. Given that previous research has indicated a link between blood lipid levels and inflammation [39], we further adjusted for blood lipid parameters and found that the serum sASGR1 concentration was significantly positively correlated with the serum hs-CRP level (*β* = 0.86, *P* < 0.001), WBC count (*β* = 0.13, *P* = 0.004), and neutrophil count (*β* = 0.10, *P* = 0.008). Additionally, a similar association was observed between sASGR1 concentration and monocyte count (*β* = 0.009, *P* = 0.014).

**Table 3:** Association of sASGR1 with hs-CRP levels, WBC counts, and WBC subsets according to the different models

Models	Crude Model		Model I		Model II		Model III	
	*β*	*P*	*β*	*P*	*β*	*P*	*β*	*P*
	(95% CI)		(95% CI)		(95% CI)		(95% CI)	
hs-CRP	0.99	< 0.001	1.11	< 0.001	1	< 0.001	0.86	< 0.001
	(0.56, 1.42)		(0.79, 1.43)		(0.67, 1.33)		(0.51, 1.20)	
WBC	0.17	0.002	0.17	< 0.001	0.12	0.004	0.13	0.004
	(0.06, 0.27)		(0.09, 0.26)		(0.04, 0.21)		(0.04, 0.22)	
Neutrophil	0.14	0.002	0.14	< 0.001	0.09	0.011	0.1	0.008
	(0.05, 0.24)		(0.07, 0.22)		(0.02, 0.16)		(0.03, 0.18)	
Monocyte	0.01	0.004	0.01	0.001	0.009	0.006	0.009	0.014
	(0.00, 0.02)		(0.00, 0.02)		(0.00, 0.02)		(0.00, 0.02)	
Lymphocyte	0.008	0.575	0.01	0.302	0.01	0.392	0.01	0.577
	(-0.02, 0.04)		(-0.01, 0.04)		(-0.02, 0.04)		(-0.02, 0.04)	

The multivariate regression stepwise models are shown. hs-CRP levels, WBC counts, and WBC subsets were the dependent variables. Model I was adjusted for age, sex and BMI; Model II was adjusted for Model I plus smoking status, hypertension, T2DM incidence, statin use, and CAD. Model III was adjusted for Model II plus TG, LDL-C and HDL-C

### Subgroup analysis of the correlation between sASGR1 and inflammatory marker levels

According to the interaction test (Table 4), the effect of sASGR1 on the hs-CRP level was significantly affected by age (*p* for interaction = 0.015), sex (*p* for interaction = 0.007), BMI (*p* for interaction = 0.002), and hypertension status (*P* for interaction = 0.019), indicating that the association between the sASGR1 concentration and the hs-CRP level differed according to these variables. Notably, even after we adjusted for factors such as age, sex, BMI, statin use, smoking status, and lipid parameters, the positive correlation between sASGR1 and hs-CRP levels was still significant in patients with CAD (*β* = 0.8, *P* < 0.001). Among patients without CAD, this correlation was not observed (*P* = 0.231).

**Table 4:** Subgroup analysis of the association between sASGR1 and hs-CRP

Variables	Effect size (95% CI)	*P* value	*P* for interaction
Age			
Below 60	0.4 (-0.0, 0.9)	0.07	0.015
Over 60	1.2 (0.7, 1.8)	< 0.001	
Sex			
Male	0.5 (0.1, 0.9)	0.028	0.007
Famale	1.3 (0.8, 1.9)	< 0.001	
BMI			
<24	1.2 (0.7, 1.7)	< 0.001	0.002
>=24	0.2 (-0.4, 0.7)	0.547	
Statin use			
No	1.0 (0.5, 1.4)	< 0.001	0.110
Yes	0.4 (-0.1, 1.0)	0.103	
CAD			
No	0.7 (-0.5, 1.9)	0.231	0.980
Yes	0.8 (0.4, 1.1)	< 0.001	
Hypertension			
No	0.5 (0.0, 0.9)	0.037	0.019
Yes	1.2 (0.7, 1.7)	< 0.001	
T2DM			
No	0.9 (0.5, 1.3)	< 0.001	0.084
Yes	0.3 (-0.4, 0.9)	0.443	

Note 1: The above model was adjusted for age, sex, BMI, statin use, smoking status, hypertension, T2DM incidence, CAD status, TG, TC, LDL-C and HDL-C

Note 2: For each patient, the model was not adjusted for the stratification variable

Similarly, we also conducted subgroup analysis and interaction testing to determine the relationship between sASGR1 and WBC count. Age was the sole significant factor influencing the association between sASGR1 level and WBC count, in contrast to hs-CRP (*P* for interaction < 0.001), as shown in Table 5. Interestingly, similar to that of hs-CRP, a positive correlation between sASGR1 and WBC was observed only in CAD patients (*β* = 0.1, *P* = 0.029).

**Table 5:** Subgroup analysis of the association between sASGR1 and WBC count

Variables	Effect size (95% CI)	*P* value	*P* for interaction
Age			
Below 60	-0.0 (-0.1, 0.1)	0.794	< 0.001
Over 60	0.3 (0.2, 0.5)	< 0.001	
Sex			
Male	0.1 (-0.0, 0.2)	0.108	0.425
Female	0.2 (0.0, 0.3)	0.038	
BMI			
<24	0.2 (0.0, 0.3)	0017	0.373
>=24	0.1 (-0.1, 0.2)	0.311	
Statin use			
No	0.1 (0.0, 0.3)	0.045	0.791
Yes	0.1 (-0.0, 0.2)	0.167	
CAD			
No	0.2 (-0.2, 0.5)	0.326	0.771
Yes	0.1 (0.0, 0.2)	0.029	
Hypertension			
No	0.1 (-0.1, 0.2)	0.314	0.089
Yes	0.2 (0.1, 0.3)	0.006	
T2DM			
No	0.1 (0.0, 0.3)	0.008	0.159
Yes	0.0 (-0.2, 0.2)	0.902	

Note 1: The above model was adjusted for age, sex, BMI, statin use, smoking status, hypertension, T2DM incidence, CAD status, TG, TC, LDL-C and HDL-C.

Note 2: For each patient, the model was not adjusted for the stratification variable

## Discussion

According to our study, sASGR1 is independently and positively correlated with hs-CRP and WBC. According to our subgroup analysis, this relationship existed only for CAD patients. In addition, serum sASGR1 levels are elevated in CAD patients. sASGR1 is independently correlated with the risk of CAD after adjusting for confounding variables, and it has better diagnostic value than APO-B and LDL-C.

Since 2016, ASGR1 has received widespread attention from researchers due to its involvement in liver cholesterol metabolism [22]. Recent animal studies have shown that inhibiting ASGR1 on the hepatocyte membrane (named hASGR1) is expected to become a new strategy for reducing LDL-C levels [21]. Similarly, genetically mimicked ASGR1 inhibitors were associated with lower cholesterol levels and CAD risk [40]. In addition, an observational study showed that ASGR1 mRNA levels in peripheral blood mononuclear cells were lower in CAD patients than in non-CAD patients, but the underlying mechanism is still unknown [23]. However, there is currently no research on the role of serum sASGR1 in CAD patients. sASGR1 and LDL-C levels were found to be positively correlated in our recent study [31]. As expected, this study showed an increase in sASGR1 levels in CAD patients, which is consistent with the findings of a recent study [41]. In the present study, plasma proteomics analysis revealed ASGR1 to be a risk factor for ischemic heart disease. Thus, the results of our study lend support for sASGR1 as a potential biomarker for CAD. We also found that, after controlling for confounding variables, sASGR1 remained an independent risk factor for CAD (Table 2). As mentioned earlier, hASGR1 regulates liver cholesterol homeostasis by binding to circulating asialoglycoproteins, ultimately affecting plasma cholesterol levels and the risk of CAD [21, 22]. Previous research revealed that while sASGR1 inhibits the binding of circulating asialoglycoproteins to hASGR1, it still binds to asialoglycoproteins and enters the liver as a complex [30, 31]. Taken together, these findings imply that the entry of the sASGR1-asialoglycoprotein complex into hepatocytes may exert similar downstream biological effects as the binding of the asialoglycoprotein to hASGR1. Another hypothesis, however, is that the serum sASGR1 concentration might reflect the hASGR1 protein level. Given this, it will be intriguing to investigate whether blocking sASGR1 can lower plasma cholesterol and the risk of CAD in a manner similar to inhibiting hASGR1.

We evaluated the relationship between serum sASGR1 levels and the severity of CAD. Although the serum sASGR1 concentration was associated with the number of coronary artery lesions (Fig. 2A), the level of sASGR1 did not significantly increase as the Gensini score increased (Fig. 2B). The serum sASGR1 concentration has been reported to be positively correlated with LDL-C levels [31], but the correlation between LDL-C level and Gensini score is not significant [42, 43]. The relationship between serum sASGR1 levels and the severity of CAD in this study remains uncertain, even though the Gensini score is a more accurate indicator of plaque burden and CAD severity than the number of vascular lesions. More precise methods, such as intravascular ultrasound and optical coherence tomography, may be needed to evaluate the relationship between sASGR1 and the severity of CAD. We failed to find that sASGR1 could be used to identify individuals who had an AMI (Fig. 2C). Our previous study showed that there is no correlation between the serum troponin T concentration and sASGR1 level [31], which is consistent with the results of this study, indicating that the sASGR1 level is not related to the degree of myocardial injury.

We analyzed the diagnostic value of the sASGR1 level for CAD. Although it does not have the same diagnostic efficacy as hs-CRP, it is superior to LDL-C and APO-B. In addition, sASGR1 has advantages over hs-CRP (47.1%), APO-B (41.2%), TG (62.4%), and TC/HDL-C (69.4%) due to its relatively high diagnostic sensitivity (77.6%), indicating an advantage in the early screening of CAD.

In addition to its clear association with cholesterol metabolism, a small number of studies have shown that ASGR1 is associated with systemic inflammation. The most direct evidence shows that knockdown of *Asgr1* in mouse liver and monocytes suppressed the expression of plasma inflammatory cytokines [interleukin-1 (IL-1), IL-6, and tumor necrosis factor (TNF-α)] [27]. However, these studies on the relationship between ASGR1 and systemic inflammation have focused mainly on the role of ASGR1 as a hepatocyte or monocyte membrane receptor. The relationships between serum sASGR1 levels and inflammatory marker levels in healthy individuals and patients with disease are unknown. Our findings showed that the serum sASGR1 concentration is positively correlated with inflammatory marker levels (hs-CRP and WBC), which supports and propels previous basic research. However, the causal link between sASGR1 and inflammatory markers warrants further exploration.

Inflammation is a key factor in the development of atherosclerosis and CAD. As a stable and reliable inflammatory marker, CRP has been shown to upregulate the expression of adhesion molecules and monocyte chemokines, inhibit the production of endothelial nitric oxide synthase (eNOS), and promote arterial thrombosis, indicating direct involvement in the occurrence of atherosclerosis [44–46]. Therefore, evaluating hs-CRP levels is highly important for CAD patients without hypercholesterolemia, as there is no urgent demand for lipid-lowering agents. The CANTOS study identified for the first time the ability of anti-inflammatory treatment to reduce cardiovascular events, which was independent of blood lipid levels [47]. An association between plasma CRP and LDL-C levels has been demonstrated in previous research [39]. Despite the fact that ASGR1 does affect circulating cholesterol levels, our subgroup analysis results showed that sASGR1 remains an independent influencing factor for hs-CRP and WBC count among individuals with CAD even after adjusting for lipid parameters (Table 3). Notably, patients without CAD did not exhibit this association (Tables 4 and 5). Thus, our findings suggest a potential connection between serum sASGR1 and inflammation in patients with CAD, independent of lipid metabolism disorders. This hypothesis has actually received indirect support from earlier studies. For instance, sialylation mediated by α2,3-sialyltransferases has been linked to the recruitment of circulating inflammatory myeloid cells to the atherosclerotic vascular endothelium [22, 48, 49]. Moreover, sASGR1 levels and monocyte counts were positively correlated according to the multivariate regression model (model III: *β* = 0.009, *p* = 0.014). ASGR1 is expressed in peripheral blood monocytes [50]. These findings suggest that, in addition to being secreted by hepatocytes, serum sASGR1 may also be secreted by monocytes. In summary, the positive correlation between the serum sASGR1 concentration and inflammatory marker (hs-CRP and WBC) levels in CAD patients supports the current view that ASGR1 is a risk factor for CAD. In addition, our research provides a reference for revealing the potential role of sASGR1 in the inflammation of atherosclerosis.

### Study strengths and limitations

This is the first study in which we investigated the relationship between serum sASGR1 levels and CAD incidence, as well as inflammatory marker levels. This study has several limitations. First, this was a cross-sectional investigation, and the causal relationships between the serum sASGR1 concentration and CAD incidence or inflammatory marker levels could not be determined. However, further studies are needed to reveal the role of ASGR1 in the inflammation of atherosclerosis. Second, a relatively small sample size may hinder the ability to identify minute differences, even though the results of sample size calculations show that our sample size is appropriate for the main purpose of the study. These findings need to be confirmed in larger-sample studies. Third, the method of dividing the patients into groups with and without CAD may have resulted in selection bias. Fourth, we did not perform Western blot analysis of the serum sASGR1 concentration to confirm that this parameter is a biomarker for CAD, as suitable antibodies were not found. Finally, we did not investigate the correlation between sASGR1 levels and the levels of other inflammatory cytokines, such as ILs.

## Conclusions

Our study suggested that serum sASGR1 levels are elevated in CAD patients and may be an independent risk factor for CAD. Moreover, sASGR1 was independently and positively correlated with inflammatory marker levels in CAD patients, even after controlling for lipid parameters. The potential role of sASGR1 in inflammation in atherosclerosis, independent of cholesterol metabolism, may need further study.

## Data Availability

The data that support the findings of this study are available from the corresponding author upon reasonable request.

## Abbreviations

ASCVD: Atherosclerotic cardiovascular disease
ASGR1: Asialoglycoprotein receptor 1
CAD: Coronary artery disease
sASGR1: Soluble ASGR1
hASGR1: Hepatic ASGR1
Hs-CRP: High-sensitivity CRP
WBCC: White blood cell count
LDL-C: Low-density lipoprotein cholesterol
HDL-C: High-density lipoprotein cholesterol
VCAM: Vascular cell adhesion molecule-1
ICAM: Intercellular adhesion molecule-1
MCP-1: Monocyte chemotactic protein-1
APO-B: Apolipoprotein B
APO-A1: Apolipoprotein A1
TG: Triglyceride
TC: Total cholesterol
Lp(a): Lipoprotein (a)
IL-1: Interleukin-1
TNF-α: Tumor necrosis factor
EGFR: Epidermal growth factor receptor
AMI: Acute myocardial infarction
T2DM: Type 2 Diabetes Mellitus
BMI: Body mass index
ROC: Receiver operating characteristic
AUC: Area under the curve
ELISA: Enzyme-linked immunosorbent assay
CI: Confidence interval
